# Supplementary material for: Hybrid Models and Biological Model Reduction with PyDSTool
Source: PLoS Comput Biol. 2012 Aug 9;8(8):e1002628. doi: 10.1371/journal.pcbi.1002628 (PMC3415397; doi:10.1371/journal.pcbi.1002628)
Supplement: Text S4 — Complete source code for the PyDSTool package (version 0.88.120504). Includes API documentation and help files linking to web pages. This file is identical to the current public release on Sourceforge.net. (ZIP) [file pcbi.1002628.s004.zip › PyDSTool/html/toc-PyDSTool.Trajectory'-module.html]

xml version="1.0" encoding="ascii"?


Trajectory'


# Module Trajectory'

---

## Classes

HybridTrajectory  
 Trajectory  

## Functions

convert\_ptlabel\_events  
 findApproxPeriod  
 numeric\_to\_traj  
 pointset\_to\_traj  

## Variables

API

Continuous

Discrete

Inf

LargestInt32

NAMESEP

ONES

ZEROS

\_1DimplicitSolveMethods

\_all\_complex

\_all\_float

\_all\_int

\_all\_numpy\_complex

\_all\_numpy\_float

\_all\_numpy\_int

\_complex\_types

\_float\_types

\_implicitSolveMethods

\_indentstr

\_int\_types

\_num\_equivtype

\_num\_maxmin

\_num\_name2equivtypes

\_num\_name2type

\_num\_type2name

\_num\_types

\_pytypefromtype

\_real\_types

\_seq\_types

builtinFnSigInfo

builtin\_auxnames

contained

name\_chars\_RE

notcontained

null\_predicate

num\_chars

protected\_allnames

protected\_auxnamesDB

protected\_macronames

protected\_mathnames

protected\_randomnames

protected\_scipynames

protected\_specialfns

syms

targetLangs

uncertain

---

[hide private]
